# What kinds of policies to reduce health inequalities in the UK do researchers support?

**DOI:** 10.1093/pubmed/fdu057

**Published:** 2014-08-30

**Authors:** Katherine E. Smith, Mor Kandlik Eltanani

**Affiliations:** 1 Global Public Health Unit, School of Social and Political Science, University of Edinburgh, Edinburgh EH8 9LD, UK; 2 Sociology, School of Social and Political Science, University of Edinburgh, Edinburgh EH8 9LD, UK

**Keywords:** evidence-based policy, health inequalities, social determinants

## Abstract

**Background:**

Despite a wealth of research and policy initiatives, progress in tackling the UK's health inequalities has been limited. This article explores whether there appears to be consensus among researchers about the kinds of policies likely to reduce health inequalities.

**Methods:**

Ninety-nine proposals for addressing health inequalities were identified from multiple sources. Forty-one researchers participated in a survey assessing the extent to which they believed each proposal would reduce health inequalities, based on three criteria. The 20 proposals generating most support were employed in a second stage, in which 92 researchers indicated which proposals they felt would have the *greatest* impact on reducing health inequalities.

**Results:**

Some consensus exists among researchers about the policy approaches likely to reduce UK health inequalities: a more progressive distribution of income/wealth, greater investment in services for deprived communities, plus regulatory policies to limit the impact of lifestyle-behavioural risks. However, researchers' support for proposals varies depending whether they are asked to express their expert opinion or to comment on the strength of the available evidence.

**Conclusions:**

When consulting researchers about health inequalities, policymakers need to consider whether they are seeking research-informed expertise *or* assessments of the available evidence; these questions are likely to yield different responses.

## Introduction

A wealth of health inequalities research has been produced over the past 40 years, much of it within the UK,^1–5^ where, since 1997, reducing health inequalities has been a stated policy priority.^6^ Yet, despite being positioned as an international leader in efforts to reduce health inequalities,^7^ the UK's health inequalities have reportedly continued to widen.^8–10^ This has been partially attributed to the lack of a public mandate for the ‘upstream’, socio-economic policies that many studies suggest are required.^7,9,11^ This has prompted calls for more public health advocacy to ensure that future policymakers have a clearer public mandate for pursuing the kinds of policies supported by the available evidence.^9,12^ Effective public health advocacy requires consensus around clear policy objectives.^13^ Yet, despite multiple reflections on the UK's efforts to reduce health inequalities,^8,9,11,12,14^ there has been little attempt to examine what advocating for more egalitarian policies means in practical terms.^15^

A recent report outlines nine proposals from high-profile researchers who were asked to recommend one intervention to reduce health inequalities (to be implemented at a local-level).^16^ This report is an innovative attempt to encourage researchers to make their recommendations for action to reduce health inequalities more explicit, but it does not reveal whether there is any consensus within the broader research community around any of the nine proposals. In contrast, this article draws on a two-stage online survey to specifically examine the level of consensus among researchers around potential policies for tackling health inequalities in the UK. Although health inequalities research is often depicted as fractured,^17–20^ the findings suggest that there are clear areas of consensus among researchers as to the kinds of policies likely to reduce health inequalities. However, researchers respond differently when asked what they, as research experts, believe will be likely to reduce health inequalities and when asked about the strength of available evidence.

## Methods

In December 2012, a symposium exploring the future of health inequalities research took place in Scotland, attended by 87 individuals involved in research, policy, practice and/or advocacy relating to health inequalities, from across the UK. Individuals were invited based on the assessment of a Steering Group consisting of health inequalities researchers from a range of disciplines (geography, medicine, political science, sociology and social policy). Discussions were informed by the findings of a large qualitative study exploring the relationship between health inequalities research and policy.^20^ The aim was to involve individuals with relevant expertise working in a variety of institutional settings, with a range of disciplinary and methodological expertise. Symposium discussions reinforced a perception, documented elsewhere,^20^ that there is a lack of consensus around policy objectives among health inequalities researchers. An online survey was developed to examine the accuracy of this perception.

### Constructing the online survey

Individuals who had been invited to the symposium, or who had expressed an interest in the event, were subsequently invited (via email) to propose policies that they felt would reduce health inequalities. The lead author also searched post-1997 health inequalities research literature for reports and papers identifying clear policy proposals for reducing population-level health inequalities in the UK^1,4,21,22^ and identified proposals for tackling health inequalities put forward by the 112 individuals interviewed in a previous qualitative study.^20^ Collectively, this approach yielded well over 100 policy proposals. By merging relatively similar proposals, the list was reduced to 99. These proposals were organized into 10 thematic policy clusters: A two-stage online survey involving these 99 proposals was subsequently developed, using a SurveyExpression's survey design tool. The survey was pre-tested with four researchers.

1. income and wealth (15 proposals);
2. employment and training (10 proposals);
3. housing, environment and transport (11 proposals);
4. alcohol and illicit drugs (9 proposals);
5. nutrition (10 proposals);
6. tobacco (7 proposals);
7. health education and community assets (6 proposals);
8. early years, youth and education (12 proposals);
9. health and other public services (13 proposals) and
10. the policymaking process (6 proposals).

### Stage 1 of the online survey

The first stage of the survey asked researchers to indicate their level of agreement with each of the 99 policy proposals according to the three sets of criteria summarized in Box 1. A five-point Likert scale which ranged from ‘strongly disagree’ to ‘strongly agree’ was employed for each question. At this stage, participants were not asked to comment on the *extent* to which they believed each policy proposal was likely to reduce health inequalities. Participation was on an anonymous basis and respondents were allowed to leave responses blank. Supplementary data Appendix 1 summarize key information about the researchers involved the first stage of the survey, based on responses to questions concerning their personal and professional characteristics.

Box 1 **The three statements researchers were asked to consider for each of the 99 policy proposals included in the first stage of the survey**

1. Based purely on my expert opinion (i.e. not taking into account what is socially, politically or economically feasible) I believe this suggestion would reduce population-level health inequalities in the UK

2. I believe that the ability of this suggestion to reduce health inequalities is strongly supported by available evidence

3. Taking into account the current social, political and economic context, I believe that this is an appropriate policy recommendation for the health inequalities research community to make

The approach to identifying potential participants in the survey mirrored that for the symposium (see above) with the additional criteria that participants had to be involved in *research* relating to health inequalities in the UK. A snowball element was incorporated with participants being asked to suggest additional potential participants. In total, 124 researchers were sent a personal request to complete this part of the survey (by email) and 41 researchers did so. This represents a response rate of 33%. As this stage of the survey took 30–60 min to complete (according to participants), this was deemed a good response rate.

### Stage 2 of the online survey: prioritization of the most popular policy proposals

In analysing the results from the first stage of the survey, we ranked the level of support for each policy proposal using each of the three statements respondents were asked to consider (Box 1) (i.e. we created three, ranked lists). We then took the 12 policy proposals which received the most support on each list and combined these proposals to create a list of the ‘most popular policy proposals’. After removing duplicates, this list contained 20 policy proposals. This list was employed for a second stage of the survey, in which respondents were each asked to divide 100 points between the ‘top twenty’ policy proposals, allocating more points to the proposals they believed would have the *greatest* impact on reducing health inequalities at the population level (taking relative and absolute measures of health inequalities into account^1^). Participants were allowed to allocate their 100 points in any way they liked, although they had to use all 100 points in order to submit their response.

An invitation to complete this second stage of the survey was sent to all of the individuals who were asked to complete the first stage (with no requirement that participants had to have completed the first stage to participate) and forwarded by some participants to colleagues. It was also distributed around relevant email groups, including the Health Equity Network, the Politics of Health Group, the Social Policy Association jiscmail, the Social Medicine Association email group, the five UKCRC centres of public health excellence and the Public Health Information Network for Scotland. More than twice as many researchers (*n* = 92) completed the second part of the survey. This is likely to be partly because the invitation was circulated to a larger number of people and partly because it was much shorter (pre-testing indicated that it took ∼10 min to complete).

## Results

### What policy proposals for reducing health inequalities did participants support?

Table 1 provides a summary of the ‘top 10’ (i.e. most supported) policy proposals for *each* of the three statements in Box 1 (the colour-coding reflects the ‘thematic’ policy clusters used to organize the 99 policy proposals in the survey—see Supplementary data Appendix 1). The results highlight that implementing a more progressive taxation and benefits system was consistently the most popular proposal, across all three statements in Box 1. However, beyond this, the policy preferences of respondents varied for the three statements.

**Table 1:** The 10 policy proposals receiving the most support from participants for each of the statements in Box 1

*Question from Box 1*	*Policy proposal*	*% disagree/strongly disagree*	*% agree/strongly agree*	*Total no. who answered question*
Row A - 1. ‘expert opinion’	• Review and implement more progressive systems of taxation, benefits, pensions and tax credits that provide greater support for people at the lower end of the social gradient and do more to reduce inequalities in wealth	5.0	92.5	40
	• Develop and implement a minimum income for healthy living	7.7	92.3	39
	• Invest more resources in support for vulnerable populations, by providing better homeless services, mental health services etc	0	91.7	36
	• Invest more resources in active labour market programmes to reduce long-term unemployment	2.5	90.0	40
	• Invest more resources in primary care health services serving very deprived areas	2.6	89.5	38
	• Support an enhanced home building programme and invest in decent social housing to bring down housing costs	4.9	87.8	41
	• Increase the national minimum wage	10.0	87.5	40
	• Reduce speeds in urban areas, starting with the poorest areas (20 m.p.h. is plenty)	7.5	87.5	40
	• Increase social protection for those on the lowest incomes and provide more flexible income and welfare support for those moving in and out of work (‘flexicurity’)	5.1	87.2	39
	• Increase the proportion of overall government expenditure allocated to the early years and ensure this expenditure is focused progressively across the social gradient	0	87.2	39

Row B - 2. ‘strongly supported by available evidence’	• Review and implement more progressive systems of taxation, benefits, pensions and tax credits that provide greater support for people at the lower end of the social gradient and do more to reduce inequalities in wealth	5.0	85.0	40
	• Fluoridate domestic water supplies (where this is not already done)	2.8	77.8	36
	• Provide stop-smoking services with additional targeting within poorer communities	0	74.3	35
	• Increase the price of tobacco products via tax increases	8.3	72.2	37
	• Increase social protection for those on the lowest incomes and provide more flexible income and welfare support for those moving in and out of work (‘flexicurity’)	5.1	71.8	39
	• Reduce speeds in urban areas, starting with the poorest areas (20 m.p.h. is plenty)	10.3	71.8	39
	• Reduce the availability of tobacco products (both legal and illicit)	5.7	71.4	35
	• Introduce standardized packaging of tobacco products (i.e. remove branding)	2.9	70.6	34
	• Maintenance (and improvement) of the NHS in a recognizable form	5.9	70.6	34
	• Introduce a minimum price for alcohol products via MUP (minimum unit pricing)	7.5	70.0	40
Row C - 3. ‘appropriate policy recommendation for the health inequalities research community to make’	• Review and implement more progressive systems of taxation, benefits, pensions and tax credits that provide greater support for people at the lower end of the social gradient and do more to reduce inequalities in wealth	4.88	87.80	41
	• Develop and implement a minimum income for healthy living	10.00	85.00	40
	• Provide stop-smoking services with additional targeting within poorer communities	0	83.78	37
	• Invest more resources in support for vulnerable populations, by providing better homeless services, mental health services etc	5.56	83.33	36
	• Invest more resources in active labour market programmes to reduce long-term unemployment	5.00	82.50	40
	• Introduce a minimum price for alcohol products via MUP	2.50	82.50	40
	• Invest more resources in primary care health services serving very deprived areas	2.70	81.08	37
	• Introduce standardized packaging of tobacco products (i.e. remove branding)	2.86	80.00	35
	• Reduce speeds in urban areas, starting with the poorest areas (20 m.p.h. is plenty)	7.50	80.00	40
	• Increase the national minimum wage	9.76	78.05	41
				

m.p.h., miles per hour; MUP, minimum unit pricing.

The biggest difference within Table 1 is between Row A (expert opinion) and Row B (strength of available evidence); the former features four economic proposals and *no* proposals around lifestyle-behavioural interventions, while the latter includes six proposals relating to reducing lifestyle-behavioural risk factors, three of which are tobacco-focused. Row C falls in between, with three policy proposals relating to lifestyle-behavioural interventions (all of which also appear in Row B). However, the three proposals which appear in Row B but not C (fluoridating domestic water supply; increasing the price of tobacco products via tax increases and reducing the availability of tobacco products) did still all score relatively highly when respondents were asked to focus on ‘appropriate’ policy recommendations, falling only just outside the top 10. This suggests that respondents tended to believe that lifestyle-behavioural interventions were both better supported by available evidence and more politically and socially ‘feasible’ than the kinds of ‘upstream’, economic policies they felt were more likely to be effective.

Only three policy proposals feature in Row A *and* Row B. This indicates that researchers provide markedly different responses to the questions: (i) what do *you* think would be effective in reducing health inequalities? and (ii) what do you think the *available research evidence* suggests would be effective in reducing health inequalities? It is also notable that the combined percentages for ‘agree’ and ‘strongly agree’ are consistently higher in Row A (ranging from 87.18 to 92.5%) than they are in Row B (which ranges from 70 to 85%). This suggests that there is a greater consensus among researchers about the kinds of policy proposals that they themselves believe are likely to reduce health inequalities than there is for their sense of the strength of the available evidence.

Free-text comments and email feedback from participants suggest that, of the three statements in Box 1, respondents were least clear how to respond when asked to say whether they felt policy proposals would be ‘an appropriate policy recommendation for the health inequalities research community to make’ (Row C). For some, this was attributed to an uncertainty about how to interpret the word ‘appropriate’, while others challenged the notion that an identifiable ‘health inequalities research community’ exists. The answers respondents provided seem to fall somewhere in between Rows A and B (e.g. there is more consensus evident in Row C than in Row B (evidence) but less than Row A (expert opinion)).

Reflecting the proposals in Table 1, the list of 20 proposals employed in the second stage of the survey (Table 2) included a mix of upstream, socio-economic policies, lifestyle-behavioural interventions and public sector, service-orientated interventions. The results from the second stage (Table 2) provide a further illustration of what appears to be a clear consensus among researchers that upstream, socio-economic policies are likely to have the greatest impact on reducing health inequalities. The proposal to ‘review and implement more progressive systems of taxation, benefits, pensions and tax credits’ was both the most frequently endorsed (75 out of 92 respondents were supportive of it) and the proposal which received the largest number of averaged points (17.4 out of 100 potential points). More broadly, four of the top five policy proposals (as measured by average number of points allocated) concern income/wealth and the other focuses on progressively distributing government expenditure on early years. Although seven of the ‘top 20’ policy proposals involve reducing lifestyle-behavioural risk factors (four of which are tobacco related), these proposals are all relatively upstream (e.g. the most popular of these involved protecting the policy process from commercial interests) and, on average, received a low number of points. This suggests that, although researchers believe there is good evidence that these kinds of lifestyle-behavioural-related interventions will reduce health inequalities, there is a consensus that these kinds of responses are likely to have a relatively limited impact.

**Table 2:** The results of the second stage of the survey, in which participants were asked to distribute 100 points according to the policy proposals they felt would be likely to have the greatest impact on reducing health inequalities in the UK

*Policy proposal*	*Mean point for all participants (rank by this measure)*	*Number of participants who allocated points to the policy (rank by this measure)*
• Review and implement more progressive systems of taxation, benefits, pensions and tax credits that provide greater support for people at the lower end of the social gradient and do more to reduce inequalities in wealth	17.4 (1)	75 (1)
• Develop and implement a minimum income for healthy living	10.1 (2)	62 (=4)
• Increase the proportion of overall government expenditure allocated to the early years and ensure this expenditure is focused progressively across the social gradient	7.5 (3)	56 (8)
• Increase social protection for those on the lowest incomes and provide more flexible income and welfare support for those moving in and out of work (‘flexicurity’)	6.8 (4)	62 (=4)
• Support an enhanced home building program and invest in decent social housing to bring down housing costs	6.5 (5)	63 (3)
• Invest more resources in state-funded education, with additional investments for schools serving more deprived communities	6.3 (=6)	65 (2)
• Introduce policies which intensively focus on improving literacy among primary school children in deprived areas through one-to-one teaching for those with low reading scores	6.3 (=6)	58 (6)
• Invest more resources in active labour market programmes to reduce long-term unemployment	5.7 (8)	57 (7)
• Invest more resources in support for vulnerable populations, by providing better homeless services, mental health services etc.	5.1 (=9)	52 (=10)
• Implement measures to protect the policy process and decision-making from interference by relevant commercial sector interests (e.g., alcohol, tobacco and ultra-processed food manufacturers and retailers)	5.1 (=9)	53 (9)
• Invest more resources in primary care health services serving very deprived areas	4.5 (=11)	52 (=10)
• Increase the national minimum wage	4.5 (=11)	49 (12)
• Maintenance (and improvement) of the NHS in a recognizable form	3.3 (13)	39 (13)
• Introduce a minimum price for alcohol products via minimum unit pricing	2.6 (14)	37 (14)
• Increase the price of tobacco products via tax increases	1.7 (15)	34 (=15)
• Reduce the availability of tobacco products (both legal and illicit)	1.7 (16)	34 (=15)
• Provide stop-smoking services with additional targeting within poorer communities	1.4 (17)	28 (18)
• Reduce speeds in urban areas, starting with the poorest areas (20 m.p.h. is plenty)	1.4 (18)	31 (17)
• Introduce standardized packaging of tobacco products (i.e. remove branding)	1.1 (19)	26 (19)
• Fluoridate domestic water supplies (where this is not already done)	1.0 (20)	25 (20)

### What were the least popular policy proposals for reducing health inequalities?

Table 3 summarizes the policy proposals which were least supported by researchers as means of reducing health inequalities in the UK, for each of the three statements in Box 1. Overall, the percentages in Table 3 are relatively low and, in most cases, a majority of respondents agreed these policy proposals would be effective at reducing health inequalities. Only four of the 99 policy proposals included in the first stage of the survey were categorically not supported as means of tackling health inequalities by more than half of the respondents for at least one of the three statements in Box 1. These four proposals (see* in Table 2) were diverse, suggesting health inequalities researchers are not unsupportive of using any overarching policy approach but that there are widespread concerns about some specific policy proposals. Population-wide health education campaigns such as *Change4Life*, introduced by Labour in England in 2009^23^ and still sponsored by the current government, was the only policy proposal that more than half of respondents consistently disagreed would be likely to reduce health inequalities across all three statements in Box 1.

**Table 3:** The 10 policy proposals which participants regarded as least likely to reduce health inequalities, based on the three criteria in Box 1.

*Question from Box 1*	*Policy proposal*	*% disagree/strongly disagree*	*Total no. who answered question*
Row A - 1. ‘expert opinion’	• Continue to invest in population-wide health promotion campaigns such as Change4Life*	63.89	36
	• Implement ‘poverty mentoring’ schemes, where people in high status positions spend time with people who live in poverty	44.44	36
	• Legislate for smoke-free homes	40.00	35
	• Target long-lasting contraceptives at young women in deprived communities	37.84	37
	• Introduce a cap on public sector salaries	37.50	40
	• Introduce standarized packaging of alcohol products (i.e. remove branding)	34.21	38
	• Implement guidance on stress management and the effective promotion of wellbeing and physical and mental health at work	34.15	41
	• Increase overall NHS spending	31.58	38
	• Include socio-economic status as a protected characteristic of equalities legislation	30.77	39
	• Hypothecate (earmark/ringfence) portions of taxes on health-damaging products (e.g. tobacco, alcohol and petrol) for investment in health improvement, especially in poorer areas	30.00	40
Row B - 2. ‘strongly supported by available evidence’	• Continue to invest in population-wide health promotion campaigns such as Change4Life*	71.43	35
	• Provide subsidized fuel or fuel supplements for those on the lowest incomes to address fuel poverty*	53.85	39
	• Legislate for smoke-free homes	44.12	34
	• Implement ‘poverty mentoring’ schemes, where people in high status positions spend time with people who live in poverty	44.12	34
	• Target long-lasting contraceptives at young women in deprived communities	43.24	37
	• Introduce a cap on public sector salaries	42.50	40
	• Introduce a cap on the wealth that any one individual can inherit	41.03	39
	• Tax capital gains at the same rate as income tax	35.90	39
	• Require the highest paid employees of a company to earn no > 20 times the salary of the lowest paid employees	35.90	39
	• Introduce a national maximum income for all (including bonuses)	35.90	39
Row C - 3. ‘appropriate policy recommendation for the health inequalities research community to make’	• Continue to invest in population-wide health promotion campaigns such as Change4Life*	63.89	36
	• Legislate for smoke-free homes*	54.29	35
	• Target long-lasting contraceptives at young women in deprived communities*	51.35	37
	• Introduce a national maximum income for all (including bonuses)	42.50	40
	• Require fee-paying (private) schools to allocate at least 50% of their places for non-fee paying children living in deprived communities	40.54	37
	• Introduce a cap on the wealth that any one individual can inherit	40.00	40
	• Introduce a cap on public sector salaries	39.02	41
	• Implement ‘poverty mentoring’ schemes, where people in high status positions spend time with people who live in poverty	38.89	36
	• Increase overall NHS spending	36.84	38
	• Increase taxes on petrol	36.59	41

Reflecting the findings in section ‘What policy proposals for reducing health inequalities did participants support?’, it is notable that three lifestyle-behavioural policy proposals feature in the 10 policy proposals which participants regarded as least likely to reduce health inequalities when they were asked to respond based purely on their ‘expert opinion’ (Row A of Table 3), whereas only one lifestyle-behavioural policy proposal features in the 10 proposals for which respondents felt the evidence was least strong (Row B). Likewise, six socio-economic, upstream interventions feature in Row B (strength of evidence), whereas only one policy proposal in this genre features in Row A (expert opinion). This reinforces the idea that researchers perceive the evidence-base to be limited for some socio-economic, upstream interventions, even though they believe these kinds of policies are most likely to reduce health inequalities.

The proposals relating to income and wealth with which most respondents disagreed tended to involved capping income or wealth in some way. In contrast, the economic proposals which received the most support (see section ‘What policy proposals for reducing health inequalities did participants support?’) focused on increasing the wealth of poorer groups. This suggests that some health inequalities researchers are more concerned with poverty and deprivation than economic inequality *per se*, despite the existence of research stressing the importance of relative inequalities.^24–26^

### Which policy proposals for reducing health inequalities were the most divisive?

Table 4 considers the policy proposals that appeared to be most divisive among the researchers participating in the first part of the survey, listing the policy proposals for which over 25% of participants both disagreed/strongly disagreed and agreed/strongly agreed that it would reduce health inequalities. Table 4 suggests health inequalities researchers are more divided about proposals relating to lifestyle-behaviours when they are asked to respond based on their expert opinion (Row A) or when thinking about what it would be appropriate for the health inequalities research community to support (Row C), than when asked to respond based on strength of available evidence (Row B).

**Table 4:** The policy proposals for reducing health inequalities which appeared to be most divisive based on the three criteria in Box 1.

*Question from Box 1*	*Policy proposal*	*% disagree/strongly disagree*	*% agree/strongly agree*	*Total no. who answered question*
Row A - 1. ‘expert opinion’	• Legislate for smoke-free homes	40.00	25.71	35
	• Introduce a cap on public sector salaries	37.50	45.00	40
	• Introduce standarized packaging of alcohol products (i.e. remove branding)	34.21	34.21	
	• Implement guidance on stress management and the effective promotion of wellbeing and physical and mental health at work	34.15	34.15	41
	• Increase overall NHS spending	31.58	28.95	38
	• Include socio-economic status as a protected characteristic of equalities legislation	30.77	35.90	39
	• Hypothecate (earmark/ringfence) portions of taxes on health-damaging products (e.g. tobacco, alcohol and petrol) for investment in health improvement, especially in poorer areas	30.00	45.00	40
	• Develop and roll-out health promotion (e.g. anti-smoking) campaigns that are specifically targeted at deprived communities	28.95	34.21	38
	• Introduce a cap on the wealth that any one individual can inherit	28.21	53.85	39
	• Increase the taxes that apply to second homes, holiday homes and empty commercial property	27.50	45.00	40
	• Work to increase uptake of pharmaceutical products that reduce the risks of experiencing cardiovascular disease (e.g. statins)	26.32	34.21	38
	• Pass responsibility for reducing health inequalities to a central government office, rather than to departments/directorates of health	25.71	37.14	35
	• Introduce further national targets for reducing health inequalities	25.00	47.22	36

Row B - 2. ‘evidence supported by available evidence’	• Provide subsidized fuel or fuel supplements for those on the lowest incomes to address fuel poverty	53.85	46.15	39
	• Introduce a cap on public sector salaries	42.50	27.50	40
	• Introduce a cap on the wealth that any one individual can inherit	41.03	33.33	39
	• Require the highest paid employees of a company to earn no >20 times the salary of the lowest paid employees	35.90	38.46	39
	• Introduce a national maximum income for all (including bonuses)	35.90	38.46	39
	• Tax capital gains at the same rate as income tax	35.90	30.77	39
	• Ensure all public and private sector employers adhere to equality guidance and legislation	35.00	27.50	40
	• Introduce rent controls (reducing housing benefit bills)	30.77	48.72	39
	• Implement guidance on stress management and the effective promotion of wellbeing and physical and mental health at work	30.77	28.21	41
	• Increase the taxes that apply to second homes, holiday homes and empty commercial property	30.00	32.50	40
	• Implement a complete ban on the advertising of alcohol products	25.64	33.33	39

Row C - 3. ‘appropriate policy recommendation for the health inequalities research community to make’	• Introduce a national maximum income for all (including bonuses)	42.50	35.00	40
	• Introduce a cap on the wealth that any one individual can inherit	40.00	40.00	40
	• Introduce a cap on public sector salaries	39.02	41.46	41
	• Increase taxes on petrol	36.59	26.83	41
	• Include socio-economic status as a protected characteristic of equalities legislation	33.33	30.77	39
	• Encourage and incentivize union membership and/or the development of worker co-operatives	32.50	32.50	40
	• Work to increase uptake of pharmaceutical products that reduce the risks of experiencing cardiovascular disease (e.g. statins)	31.58	28.95	38
	• Provide targeted incentives to help poorer groups quit smoking (e.g. provide vouchers to those who are able to demonstrate evidence of quitting)	29.73	48.65	37
	• Legislate for smoke-free cars	28.57	42.86	35
	• Pass responsibility for reducing health inequalities to a central government office, rather than to departments/directorates of health	28.57	34.29	35
	• Introduce further national targets for reducing health inequalities	27.78	44.44	36
	• Hypothecate (earmark/ring-fence) portions of taxes on health-damaging products (e.g. tobacco, alcohol and petrol) for investment in health improvement, especially in poorer areas	26.83	34.15	41
	• Ensure access to higher education is affordable (e.g. by getting rid of tuition fees where they are in place)	26.32	44.74	38
	• Develop and roll-out health promotion (e.g. anti-smoking) campaigns that are specifically targeted at deprived communities	26.32	31.58	38
	• Provide free public transport for all children	25.64	41.03	39
	• Require the highest paid employees of a company to earn no >20 times the salary of the lowest paid employees	25.00	60.00	40

In relation to proposals about income/wealth redistribution, health inequalities researchers appear to be most divided when asked to respond based on the strength of the available evidence (Row B) and least divided when asked to focus on their ‘expert opinion’ (Row A). These findings are in line with those presented in sections ‘What policy proposals for reducing health inequalities did participants support?’ and ‘What were the least popular policy proposals for reducing health inequalities?’. Overall, there were only two policy proposals for which 25% or more of the participants disagreed/strongly disagreed that the proposals would reduce health inequalities, while another 25% or more agreed/strongly agreed, across all three of the statements in Box 1. Both of these proposals concern capping the wealth of some groups (introduce a cap on public sector salaries and introduce a cap on the wealth that any one individual can inherit).

## Discussion

### Main finding of this study

Previous research suggests policymakers perceive there to be a lack of agreement among health inequalities researchers regarding effective (or promising) interventions.^20^ In contrast, the results suggest that there is a fairly high degree of consensus among researchers about the kinds of policies likely to reduce health inequalities in the UK, at least when researchers are asked to provide perspectives based purely on their expert opinion. These policies, which were also the policies respondents suggested were likely to have the greatest impact (in the second part of the survey), involve more progressive distribution of income/wealth and greater investments in some public services supporting deprived/vulnerable communities (including services relating to education and the early years of life). The results reveal less about *how* researchers think a more progressive distribution of wealth should/could be achieved, although there was more consistent support for policies aiming to improve the wealth of the poorest groups (e.g. through a minimum income) than for proposals to limit the wealth of more privileged members of society. This suggests that researchers, like policymakers,^20,27^ tend to be more concerned with poverty and deprivation than inequality *per se*.^24–26^

When researchers are asked to assess policy proposals based on their sense of the strength of the available evidence or what they deem to be ‘appropriate’ recommendations for the health inequalities research community to support, they are more likely to support policy proposals intended to reduce lifestyle-behavioural risks (albeit in relatively upstream ways). The disjuncture between the kinds of evidence that the health inequalities research community is producing and researchers' perspectives on what is likely to be most effective and feasible in reducing health inequalities has been identified in previous (interview-based) studies.^20,28^ It might suggest that researchers' personal preferences are consciously informed by factors other than the available evidence (e.g. that they are ideologically or normatively driven) or, as has been argued elsewhere,^29^ that it is simply harder to establish an evidence-base for the impact of social or fiscal policies on health inequalities than it is for more individual (e.g. lifestyle-behavioural or healthcare) interventions. Reflecting this, it may be that, when asked to express a personal opinion, researchers feel comfortable drawing on broader kinds of knowledge and expertise (e.g. evidence and theories concerning the causes of health inequalities) but when asked to make a judgement that is specifically about the strength of the available evidence, they draw on particular kinds of academic work (e.g. the findings from intervention-orientated, evaluative research and systematic reviews). The latter interpretation was supported by comments made in the ‘free-text’ areas of the survey and in email feedback from participants. One respondent, for example, reflected that ‘There's lots of observational data that income is related to health but hardly any interventional data confirming that if you give people more money, their health improves. I think this is highly likely to be the case, but I don't think we can genuinely say it's strongly supported by evidence’. Others noted how difficult it can be to ‘persuade agencies to provide serious funding for the kinds of upstream interventions that would create opportunities for the generation of the evaluation evidence that is needed’. In this context, the findings suggest that efforts to ensure policies are only pursued where they are ‘evidence-based’ may be contributing to the much discussed problem of ‘lifestyle drift’ in health inequalities.^11,20,30^

### What is already known on this topic

The persistence of the UK's health inequalities, despite a wealth of research and policy activity, has prompted calls for greater advocacy to ensure public support for the kinds of policies research suggests may be necessary to reduce health inequalities.^9,20^ Public health advocacy requires developing coalitions of support around specific policy goals.^20^ Yet, it is unclear whether there is a consensus among researchers as to what is likely to work in reducing health inequalities.^20^

### What this study adds

We demonstrate that there appears to be a consensus among researchers about the need for upstream, redistributive and public-service-orientated approaches to reducing health inequalities in the UK. However, researchers' responses are notably different when asked to respond based on their personal (expert) opinion and when asked to assess the strength of the available evidence. When consulting researchers, policymakers need to be clear whether they are seeking researchers' opinions about the kinds of policies that they, as research experts, think are most likely to reduce health inequalities *or* whether they are asking researchers for an assessment of the strength of the available evidence and/or for recommendations that take account of ‘the current social, political and economic context’. Thinking about future research agendas, it seems important to do more to examine the likely impacts of the kinds of upstream policies that researchers believe are most likely to reduce health inequalities in the UK.

### Limitations of this study

It is difficult to gauge the proportion of relevant researchers involved in this survey, given the impossibility of defining a precise ‘health inequalities research community’, but the survey captured health inequalities researchers from a range of different disciplinary, institutional and geographical backgrounds/locations. Participants’ responses to the first part of the survey suggest that there may have been some bias towards: (i) researchers with an existing interest in policy analysis (perhaps enhancing their interest in participating) and (ii) researchers based in Scotland (where the background symposium was held). The second stage was widely advertised and anonymous so it is possible that some participants were not involved in health inequalities research in the UK directly.

Although we drew on multiple sources to identify policy proposals to include in the survey (and believe we captured most popular proposals), we were not able to identify *all* proposals. Several participants indicated that they would have preferred some proposals in the survey to have been worded differently and some indicated that they did not feel qualified to assess particular policy proposals.

In completing the survey, several participants commented that the design of the survey meant only four options on the five-point likert scales appeared visible on their screen (the option ‘strongly agree’ was located so far to the right-hand side of some screens that participants had to scroll to select this option). This may have caused more participants to select ‘agree’, rather than ‘strongly agree’. With this in mind, we combined ‘strongly agree’ and ‘agree’ (and ‘strongly disagree’ and ‘disagree’) for our analysis.

The order in which policy proposals appeared the survey (see Supplementary data, Appendix 2) may have impacted on variations in respondent support. For example, proposals from the ‘income and wealth’ cluster appeared first in the survey, many of which featured prominently in responses, while proposals relating to the ‘policymaking process’ appeared last in the survey and tended to attract less support.

Finally, as we did not attempt to define ‘expert opinion’, ‘available evidence’ or ‘the current social, political and economic context’ for survey participants, it is difficult to ascertain precisely how they interpreted the three questions in Box 1 and whether this varied between participants.

## Funding

This work was supported by a University of Edinburgh School of Social and Political Science Strategic Research Support fund award. The lead author (K.S.) is supported by an Economic and Social Research Council Future Research Leaders Award (grant number: ES/K001728/1).

## Supplementary Material

Supplementary Data

## Footnotes

1 Initially, the survey did not specify whether respondents should consider absolute and/or relative measures of health inequalities but, after the first seven participants completed the survey, one potential participant contacted the lead author for clarification on this issue. The survey description was subsequently amended to state that respondents should consider both relative and absolute measures of health inequalities.
