# Supplementary material for: What kinds of policies to reduce health inequalities in the UK do researchers support?
Source: J Public Health (Oxf). 2014 Aug 30;37(1):6–17. doi: 10.1093/pubmed/fdu057 (PMC4340326; doi:10.1093/pubmed/fdu057)
Supplement: Supplementary Data [file fdu057_Supplementary_Data.zip › fdu057supp_appendix1.doc]

**Web Appendix 1: The 99 policy proposals for reducing health inequalities included in the survey (suggested by participants and/or located in available research literature)**

| **Thematic cluster** | **Specific policy proposals** |
| --- | --- |
| Income and wealth | 1. **Increase the national minimum wage** 2. **Develop and implement a minimum income for healthy living** 3. **Provide subsidised fuel or fuel supplements for those on the lowest incomes to address fuel poverty** 4. **Tax capital gains at the same rate as income tax** 5. **Introduce a cap on the wealth that any one individual can inherit** 6. **Local authorities’ economic strategies should include an explicit aim to reduce income inequality gaps.** 7. **Require the highest paid employees of a company to earn no more than 20 times the salary of the lowest paid employees.** 8. **Introduce a cap on public sector salaries** 9. **Introduce a national ‘maximum income’ for all (including bonuses)** 10. **Introduce rent controls (which would also reduce housing benefit bills)** 11. Increase the taxes that apply to second homes, holiday homes and empty commercial property 12. **Extend the current council tax bands up to band Z with a view to transforming the tax into a fairer national land and property tax** 13. **Review and implement more progressive systems of taxation, benefits, pensions and tax credits that provide greater support for people at the lower end of the social gradient and do more to reduce inequalities in wealth** 14. Increase social protection for those on the lowest incomes and provide more flexible income and welfare support for those moving in and out of work ('flexicurity') 15. **Hypothecate (earmark/ring-fence) portions of taxes on health-damaging products (e.g. tobacco, alcohol and petrol) for investment in health improvement, especially in poorer areas** |
| Employment and training | 16. **Ensure all public and private sector employers adhere to equality guidance and legislation** 17. **Include socio-economic status as a protected characteristic of Equalities legislation** 18. **Implement guidance on stress management and the effective promotion of wellbeing and physical and mental health at work** 19. Encourage and incentivise employers to create or adapt jobs that are suitable for lone parents, carers and people with mental and physical health problems 20. Provide easily accessible support and advice for 16-25 year olds on life skills, training and employment opportunities 21. Provide further work-based learning for young people and those changing jobs/careers, including paid apprenticeships 22. **Encourage and incentivise union membership and/or the development of worker co-operatives** 23. **Introduce stronger employment legislation, including greater support for trades unions and restrictions on 'flexible' insecure contracts** 24. **Invest more resources in active labour market** **programmes to reduce long-term unemployment** 25. Invest in job creation (e.g. by financially supporting potential employers, particularly in areas of low employment) |
| Housing, environment and transport | 26. **Improve the energy efficiency of housing across the social gradient** 27. **Improve the quality of open and green spaces across the social gradient** 28. **Try to improve active travel, e.g. by investing in safe cycle routes and banning the creation of new parking spaces (apart from for disabled drivers) in areas where there is adequate public transport** 29. **Reduce speeds in urban areas, starting with the poorest areas (20mph is plenty)** 30. **Provide (or maintain, where already provided) free public transport for people past the retirement age (to prevent social isolation)** 31. **Provide free public transport for all children** 32. **Increase taxes on petrol** 33. **Support an enhanced home building program and invest in decent social housing to bring down housing costs** 34. **Repeal recent ‘bedroom tax’ legislation** 35. **Enhance the existing “Right to Sell” so that mortgagees have the right to stay in their home and become tenants rather than face eviction** 36. **Amend legislation so that illegal actions by landlords and banks that deprive people of their home become criminal rather than civil offences** |
| Alcohol and illicit drugs | 37. **Increase and improve the** **scale, accessibility and quality of alcohol treatment** **programmes** 38. Introduce a minimum price for alcohol products via minimum unit pricing 39. **Increase the price of alcohol products via tax increases** 40. Restrict the availability of alcohol products via further licensing restrictions 41. **Introduce further restrictions on the marketing of alcohol products (e.g. ban on TV advertising)** 42. **Implement a complete ban on the advertising of alcohol products** 43. **Introduce standarised packaging of alcohol products (i.e. remove branding)** 44. **Increase and improve the** **scale, accessibility and quality of drug treatment** **programmes** 45. **Decriminalise drug use and prioritise treatment and harm reduction (similar to Portugal)** |
| Nutrition | 46. **Provide incentives for retailers in poorer areas to promote healthier food products** 47. **Establish a local ‘food poverty partnership’ with key organisations, including health services, local authorities and voluntary organisations, to develop a local food poverty strategy** 48. Add key nutritional supplements to commonly consumed products (e.g. bread and cereals) 49. **Fluoridate domestic water supplies (where this is not already done)** 50. **Subsidise the cost of healthy (low fat, low sugar, unprocessed) food products** 51. **Ban trans fats in all foods** 52. **Provide free, nutritious school meals for all children in state schools** 53. **Restrict advertising of ultra-processed / high fat / high sugar food and drinks (e.g. introduce a ban on TV advertising before the watershed)** 54. **Implement a complete ban on the advertising of ultra-processed / high fat / high sugar food and drinks** 55. **Increase the price of ultra-processed / high fat / high sugar food and drinks via taxation** |
| Tobacco | 56. **Provide stop-smoking services with additional targeting within poorer communities** 57. **Provide targeted incentives to help poorer groups quit smoking (e.g. provide vouchers to those who are able to demonstrate evidence of quitting)** 58. **Reduce the availability of tobacco products (both legal and illicit)** 59. **Increase the price of tobacco products via tax increases** 60. **Introduce standardised packaging of tobacco products (i.e. remove branding)** 61. **Legislate for smoke-free cars** 62. **Legislate for smoke-free homes** |
| Health education and community assets | 63. **Continue to invest in population-wide health promotion campaigns such as *Change4Life*** 64. **Develop and roll-out health promotion (e.g. anti-smoking) campaigns that are specifically targeted at deprived communities** 65. **Provide area-based funding targeted at deprived communities and encourage local communities to decide how best to spend this funding on reducing health inequalities** 66. **Support locally developed community regeneration programmes that remove barriers to community participation and reduce social isolation** 67. **Invest in assets-based approaches to tackling health inequalities** 68. **Implement ‘poverty mentoring' schemes, where people in high status positions spend time with people who live in poverty** |
| Early years, youth and education (n=12) | 69. **Provide routine support to families through parenting programmes, children’s centres and key support workers** 70. **Ensure access to higher education is affordable (e.g. by getting rid of tuition fees where they are in place)** 71. **Increase the availability of non-vocational life-long learning across the life course** 72. **Increase the proportion of overall government expenditure allocated to the early years and ensure this expenditure is focused progressively across the social gradient** 73. **Invest more resources and provide more training for the childcare workforce** 74. **Invest more resources in state-funded education, with additional investments for schools serving more deprived communities** 75. **Introduce policies which intensively focus on improving literacy among primary school children in deprived areas through one-to-one teaching for those with low reading scores** 76. **Require fee-paying (private) schools to allocate at least 50% of their places for non-fee paying children living in deprived communities** 77. **Implement enhanced sex and relationship education in schools in low income areas with the aim of promoting later childbearing and more stable partnerships** 78. **Target long-lasting contraceptives at young women in deprived communities** 79. **Provide paid parental leave in the first year of life with a minimum income for healthy living** 80. **Provide means-tested, state-funded childcare (similar to Sweden)** |
| Health and other public services | 81. **Implement campaigns to tackle prejudice of various kinds (e.g. racism, sexism, homophobia and the negative characterisation of welfare recipients and people experiencing mental ill-health) nationally and in the public sector** 82. **Work to increase uptake of pharmaceutical products that reduce the risks of experiencing cardiovascular disease (e.g. statins)** 83. **Provide additional time for NHS consultations with the neediest patients** 84. **Prioritise the prevention and early detection of those conditions most strongly related to health inequalities (cancer, diabetes and cardiovascular disease)** 85. Invest more resources in primary care health services serving very deprived areas 86. **Invest more resources in support for vulnerable populations, by providing better homeless services, mental health services, etc.** 87. **Train NHS primary care staff who have regular contact with patients to respond to their patients' social circumstances which affect their health (e.g. gender-based violence, financial stress, discrimination etc.)** 88. **Require all medical students and senior health staff to spend at least two years working in a deprived community (in the UK or abroad) so that they better understand the social and material factors impacting on health** 89. **Routinely provide welfare rights and citizens advice in all health care settings** 90. **Develop stronger links between staff from area-based services (social work, mental health, addictions, child health) and general practices or groups of practices, on a named basis** 91. **Maintenance (and improvement) of the NHS in a recognisable form** 92. **Increase overall NHS spending** 93. **Fund all health care through taxation rather than through direct charges to the sick, including prescriptions, dental care and optical care** |
| The policymaking process | 94. **Introduce further national targets for reducing health inequalities** 95. **Routinely undertake health equity impact assessments on all policy in areas, including macroeconomic and fiscal policy, trade policy, foreign policy, 'defence' policy and international development** 96. **Pursue ‘Health in All Policies’ style approaches to policymaking** 97. **Pass responsibility for reducing health inequalities to a central government office, rather than to departments/directorates of health** 98. **Implement measures to protect the policy process and decision-making from interference by relevant commercial sector interests (e.g. alcohol, tobacco and ultra-processed food manufacturers and retailers)** 99. **Look to countries with good health outcomes for policy inspiration (i.e. Nordic countries) rather than ones with poor outcomes (e.g. the USA)** |
