# Supplementary material for: What kinds of policies to reduce health inequalities in the UK do researchers support?
Source: J Public Health (Oxf). 2014 Aug 30;37(1):6–17. doi: 10.1093/pubmed/fdu057 (PMC4340326; doi:10.1093/pubmed/fdu057)
Supplement: Supplementary Data [file fdu057_Supplementary_Data.zip › fdu057supp_appendix2.doc]

**Web Appendix 2**: The personal and institutional characteristics of the researchers who completed the first stage of the survey

| **Personal/professional characteristic** | **Summary of survey respondents** | |
| --- | --- | --- |
| Gender | 22 respondents identified themselves as men, 13 as women (six respondents did not specify their gender). | |
| How religious (regardless of religion)? | 21 respondents said they were not at all religious  13 respondents indicated they were somewhat religious  1 respondent indicated they were ‘very religious’ | |
| Length of time involved in research relating to health inequalities | Less than a year: 1 respondent  1-5 years: 6 respondents  6-10 years: 5 respondents  11-15 years: 6 respondents  16-20 years: 8 respondents  21-25 years: 7 respondents  26-30 years: 4 respondents | |
| Centrality of health inequalities to overall research | Central focus: 10  Important (but not the central focus): 19  Only formed a limited strand: 7 | |
| Current institutional affiliation/location | Russell group university: 22 respondents  NHS bodies: 9 respondents  Non-Russell group university: 7 respondents  Knowledge brokerage organisations: 3 respondents  Think tank : 1 respondent  (five participants either did not specify or selected ‘other’). | |
| Previous institutional affiliation/location | Non-Russell group university: 17 respondents  NHS bodies: 16 respondents  Russell group university: 14 respondents  Scottish government: 3 respondents  Charity: 3 respondents  Think tank: 3 respondents  English/UK government: 2 respondents  Knowledge brokerage organisations: 2 respondents  Local government: 1 respondent  Other: 8 respondents | |
| Geographical location (of main place of work) | Scotland: 19 respondents  England: 13 respondents  Wales: 2 respondents  Northern Ireland: 1 respondent | |
| Main geographical focus of health inequalities research | UK: 13 respondents  Scotland: 9 respondents  England: 5 respondents  Europe; 3 respondents  Wales: 2 respondents  Other: 3 respondents | |
| Main methodological approaches employed | Predominantly quantitative: 15 respondents  Predominantly mixed methods: 10 respondents  Predominantly qualitative methods: 9 respondents | |
| Disciplinary background (NB participants were asked to select all that applied) | Public health: 15 respondents  Social policy: 11 respondents  Epidemiology: 9 respondents  Sociology: 9 respondents  Medicine: 7 respondents  Political science: 5 respondents  Economics: 5 respondents  Geography: 4 respondents  Psychology: 3 respondents  History: 2 respondents  Biology: 2 respondents  Other: 3 respondents. | |
| Other research interests, in addition to health inequalities (NB participants were asked to select all that applied) | Public policy analysis: 17  Smoking/tobacco: 11  Health systems: 11  Alcohol: 10  Welfare states/welfare reform: 10  Non-communicable diseases: 9  Local environment/neighbourhoods: 8  Obesity: 8  Economics/fiscal policy: 6  Physical exercise: 6  Climate change: 5  Corporate policy influence: 5  Employment policy: 5  Housing: 5  Intelligence: 5  Food/nutrition: 4  Illicit drugs: 3  Violence: 3  Other: 5 | |
| Which political party did they feel came closest to their political views? | Labour Party: 16 respondents  Green Party; 8 respondents  Other parties: 7 respondents | |
| Educational background (at school level) | A non-selective, non-fee paying school: 17 respondents  A selective-state school: 9 respondents  A fee-paying school: 8 respondents | |
| How respondents positioned themselves on a ‘ladder’ of socio-economic position, where rung 10 represents the 10% of the UK population with the greatest wealth, the most education and the most respected jobs, and rung 1 represents the 10% of the UK population with the least wealth, least education and least respected jobs | | Rung 10 (top): 16 (45.7%)  Rung 9: 8 (22.9%)  Rung 8: 6 (17.1%)  Rung 7: 2 (5.7%)  Rung 6: 1 (2.9%)  Rung 5: 2 (5.7%)  Rungs 1-4: 0 (0%) |
| Self-declared gross pay of participants | Over £80,000: 10 respondents (30.3%)  £60,000-£79,000: 8 respondents (24.2%)  £40,000-£59,999: 9 respondents (27.3%)  £20,000-£39,999: 5 respondents (15.2%)  Under £20,000: 1 respondent (3.0%) | |
| Experience of living in ‘socially deprived’ circumstances? | 23 respondents indicated that they had never lived in what they considered to be ‘socially deprived’ circumstances.  12 respondents said that they had (although the survey was not able to capture over what kind of time period and two respondents used the free-text space to indicate it had been for a short period, with one noting it had been a personal choice). | |
